# Unraveling the mechanism of potato (*Solanum tuberosum* L.) tuber sprouting using transcriptome and metabolome analyses

**DOI:** 10.3389/fpls.2023.1300067

**Published:** 2024-01-05

**Authors:** Xiaoyuan Zheng, Mei Li, Xuejiao Zhang, Jianxin Chen, Xia Ge, Shouqiang Li, Jiachun Tian, Shilong Tian

**Affiliations:** ^1^ Agricultural Product Storage and Processing Research Institute, Gansu Academy of Agricultural Sciences, Gansu, Lanzhou, China; ^2^ College of Food Science and Engineering, Gansu Agricultural University, Lanzhou, China; ^3^ Gansu Innovation Center of Fruit and Vegetable Storage and Processing, Gansu Academy of Agricultural Sciences, Lanzhou, Gansu, China

**Keywords:** potato tuber, sprouting, mechanism, transcriptome, metabolome

## Abstract

Sprouting is an irreversible deterioration of potato quality, which leads to the production of harmful toxins and loss of the commercial value of potatoes. However, there is no report on the changes in different stages of potato sprouting through transcriptome and metabonomics. In this study, 1471 differentially expressed genes (DEGs) were found between DP and BP. In comparison with SP, a total of 6309 DEGs were detected in BP. Additionally, 6624 DEGs were identified between DP and SP. Moreover, 96 and 117 differentially accumulated metabolites (DAMs) were detected between DP and BP and between BP and SP, respectively. Furthermore, 130 DAMs were identified in total between DP and SP. In each group, a correlation analysis of DAMs and DEGs was performed to examine the regulatory network. The results indicated that the sprouting of tubers is mainly regulated by plant hormone signals, and during the sprouting of tubers, significant changes in metabolic products occur in the body. According to the combined analysis of transcriptomics and metabolomics, multiple metabolites were both positive and negative regulated by genes.

## 1 Introduction

Globally, potato (*Solanum tuberosum* L.) is the fourth largest and the third most consumed crop. These figures show how indispensable it is in the human diet and how it serves as a mainstay in the global economy (Dastmalchi et al., 2016). However, potatoes are subject to undesirable sprouting during storage. Sprouting induces significant weight loss and reduction in tuber quality. It also obstructs air movement in the potato pile, which accelerates tuber deterioration (Huang et al., 2014). In addition, during storage, sprouting of potatoes can lead to increased food wastage and a net loss for the industry (Pinhero and Yada, 2009). However, when using the tubers as seeds, it is necessary to accelerate their sprouting (Sonnewald, 2001). Thus, further studies on the mechanisms of potato germination are needed so as to better control its sprouting.

After harvesting, potato tubers enter a deep internal dormancy stage during which sprouting cannot happen even if conditions are appropriate (Draie and Abdul-Mohsen, 2021). This period begins from the moment the shoots of the plant are mowed. During dormancy, tubers can maintain their stocks of starch and protein. Moreover, biochemical and physiological processes take place in preparation for future sprouting (Ozeretskovskaya, 1990). After this stage, tuber eyes are activated and sprouts start to develop intensively, forming roots at their base (Teper-Bamnolker et al., 2010). During this phase, tubers serve as nutrition and energy source to support the sprouts’ growth (Struik, 2007). The dormancy period ends when sprouts ≥2 mm in length appear on minimum 80% of the tubers (Van-Ittersum and Scholte, 1992). Tuber sprouting is controlled by storage conditions (including temperature), tuber variety, plant hormones, genetic factors, and specific signaling molecules (Wang et al., 2020). Phytohormones play an essential role in initiating or inhibiting the dormancy of tubers. Continuous production of endogenous abscisic acid (ABA) is needed to initiate and maintain tuber sprouting. Additionally, gibberellins and ethanol may have an impact on sprouting initiation (Bajji et al., 2007). To be responsive to phytohormones, tuber cells need to be metabolically competent. This feature determines tuber sprouting as it influences the ability of phytohormones to control cell growth. During the sprouting process, potato tubers serve as a source organ that promotes the germinated sprout’s growth (Sonnewald, 2001).

There have been some studies on potato tuber sprouting before, but they have all remained at the physiological and biochemical levels. Lack of systematic and comprehensive research on the mechanism of tuber sprouting, especially based on the rapidly developing multi omics technology. In this work, tubers in the dormancy stage, early supporting stage (i.e., 160 days after the harvest), and late supporting stage were used as test materials for transcriptomic and metabolomic sequencing. This study analyzed the dynamic shifts in metabolite accumulation and gene expression in all three stages of tuber sprouting to understand the relevant mechanisms. During potato germination, several changes happen in gene expression and metabolite production in the tubers. This study aimed to offer new insights into the mechanisms of potato germination and provide solutions for a better control of tuber sprouting in future breeding and storage.

## 2 Materials and methods

### 2.1 Plant materials

Potato tubers (Atlantic, cv.) were purchased from Kaikai Potato Seed Co. Ltd., (Gansu China). Injured and infected tubers were discarded. All good tubers were stored (20 ± 3°C) for use. Tubers at the end of their dormancy stage were submerged in 1% sodium hypochlorite for 3 min for cleaning and disinfection and then washed with sterile water. After being air-dried, all tubers were stored under dark conditions (20 ± 2°C; relative humidity 75% – 85%). Each experiment was performed in three replicates, and each replicate contained 30 tubers.

### 2.2 Methods

#### 2.2.1 Sample preparation

Samples were taken at different sprouting stages (dormancy period, budding period, and sprouting period) (**Figure S1**). Five grams of tuber tissue were collected from the bud growth site (called bud eye, which is located at a depth of 1 cm under the skin) using a stainless steel punch (1 cm). The tissue was wrapped in a tin foil paper and frozen using liquid nitrogen. Subsequently, the tissue was transferred to a −80°C freezer for storage until use.

#### 2.2.2 RNA sequencing

The method described by Woolfson et al. (2018) was used to perform total RNA extraction. Use Nanodrop to detect the purity (OD260/280), concentration, and nucleic acid absorption peak of RNA, and accurately detect the integrity of RNA using Agilent 2100. Using mRNA as a template, the first cDNA strand was synthesized using six base random primers. Then, buffer, dNTPs, RNase H, and DNA polymerase I were added to synthesize the second cDNA strand. The cDNA was purified using AMPure XP beads. cDNA libraries were established according to the method described by Foucart et al. (2006) (three duplicate samples). The libraries were sequenced (with 2 × 150 paired end reads) on the platform Illumina HiSeq 2500 (purchased from Jisi Huiyuan Biotechnology Co., Ltd, Nanjing, China). The reads with high quality were applied to assemble to unigenes using the method reported by Haas et al. (2013). RNA-Seq was performed in triplicate, and the sequence mapping was done using the TopHat 2 software. Obtained sequence data were mapped for counting assemblies and a reference genome of *Solanum tuberosum* was obtained from Potato Genomics Resource (DM_1-3_516_R44_potato.v6.1, http://spuddb.uga.edu/dm_v6_1_download.shtml).

#### 2.2.3 Functional annotation

Unigenes were queried against public databases to obtain functional annotations (e < 10^−5^), including the Eukaryotic Orthologous Groups of protein (KOG), Gene Ontology (GO), UniProt, Non-redundant (Nr), and Kyoto Encyclopedia of Genes and Genomes (KEGG). KEGG pathway analyses and GO functional classifications were performed using KEGG automatic annotation servers and Web Gene Ontology Annotation Plot (WEGO), respectively.

#### 2.2.4 Quantitative reverse transcription PCR analysis

To validate the obtained results in the experiment, differentially expressed genes in plant hormone signal transduction were selected for qRT-PCR verification. qRT-PCR analysis was performed as described by Woolfson et al. (2018). The reaction mixture for RT-qPCR analysis was prepared as follows: 0.8 µL of template cDNA, 8.4 µL of RNase-free water, 0.4 µL of each primer, and 10 µL of Ultra SYBR mixture. The RT-qPCR was performed with a Roche LightCycler 480 thermocycler (rented from Roche, Bazel, Switzerland). The conditions were as follows: 95°C for 10 min, 40 cycles at 95°C for 30 s and 72°C for 30 s, followed by 1 cycle at 72°C for 10 min, and one cycle at 40°C for 30 s. The *ef1* was designed based on Nicot et al. (2005). Primer 5.0 was used to design gene-specific primer pairs, and the list of primers used in this study is shown in Supplementary Table.1. The relative expression was calculated using the 2^−ΔΔCt^ method.

#### 2.2.5 Metabolite extraction

Freeze-dried samples were pulverized in a mixer mill (45 Hz, 1 min), 100 mg of which was added to 1500 µL of methanol–water (3:1), sonicated, and homogenized at a low temperature (0°C) for 15 min. Later, samples were placed for 24 h on a shaker at 4°C and centrifuged for 15 min at 4°C (13800 *×g*). After centrifugation, the supernatant was collected and filtered with a membrane. Methanol–water (3:1) was used to dilute the filtrate two folds. The final samples were placed in glass vials. The final yield was 20 µL, which was entirely used as a QC sample for ultrahigh-performance liquid chromatography–mass spectrometry analysis (Sawada et al., 2009).

#### 2.2.6 UHPLC–MS analysis

An EXIONLC System (Sciex) was rented to perform UHPLC separation. Formic acid (0.1%) was used as mobile phase A and acetonitrile as mobile phase B. The autosampler and column temperatures were 4°C and 40°C, respectively. A Sciex QTrap 6500+ (which was purchased from Sciex Technologies) was employed in this study. The parameter settings for the ion source were: curtain gas: 35 psi; ion source gas 2: 60 psi; temperature: 400°C; ion source gas 1: 60 psi; ionspray voltage: +5500/−4500 v; and DP: ± 100 V (Zhang et al., 2015). Five duplicate samples were used for UHPLC–MS.

#### 2.2.7 Data preprocessing, annotation, and screening of differentially accumulated metabolites

Sciex Analyst Workstation Software (Version 1.6.3) was used to acquaint the data of MRM. MS raw data in the format of.wiff files were converted to TXT files using the MSconventer. In-house R database and program were utilized to perform peak detection and annotation (Kuhl et al., 2012). DAMs were identified via pairwise comparison based on a threshold: p-value ≤ 0.05 and variable importance in the projection of the PC1 of the OPLS-DA model ≥ 1.

## 3 Results

### 3.1 Analysis of differentially expressed genes

**Figure 1:**
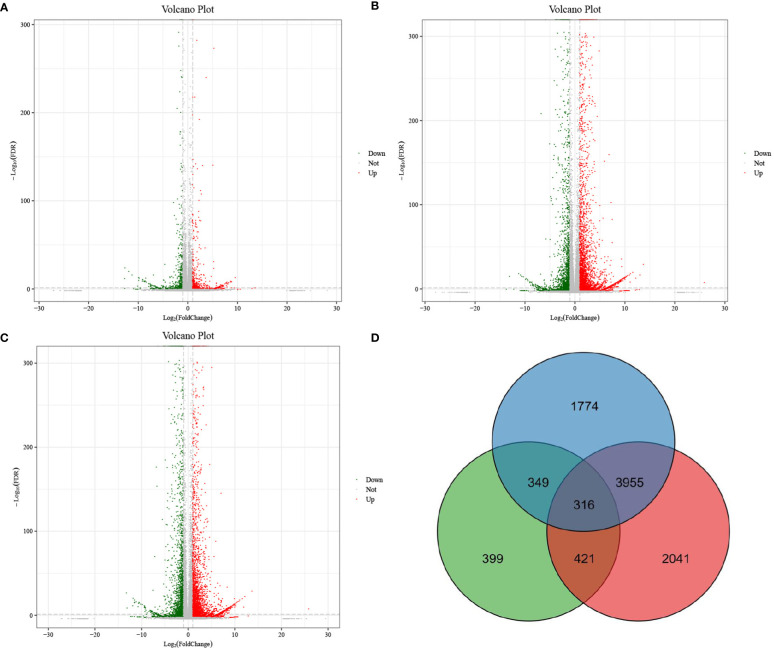
Volcano plot of differentially expressed genes between DP vs BP **(A)**, BP vs SP **(B)** and DP vs SP **(C)**. Venn diagram of differentially expressed genes between DP vs BP, BP vs SP and DP vs SP **(D)**.

Pairwise comparison was applied to screen DEGs with a threshold of false discovery rate lower than 0.05 and fold change not lower than 2. As shown in **Figure 1A**, DP and BP displayed 1471 DEGs, including 668 downregulated genes and 803 upregulated genes. As displayed in **Figure 1B**, there were 6309 DEGs between BP and SP, of which 2197 were downregulated and 4112 were upregulated. Furthermore, 6624 DEGs, including 2520 downregulated genes and 4104 upregulated genes, were found between DP and SP (**Figure 1C**). **Figure 1D** exhibits the Venn diagram for DEG screening. In all, 316 genes were found to be overlapped among the three groups of paired comparisons.

### 3.2 Differential gene KEGG enrichment analysis

**Figure 2:**
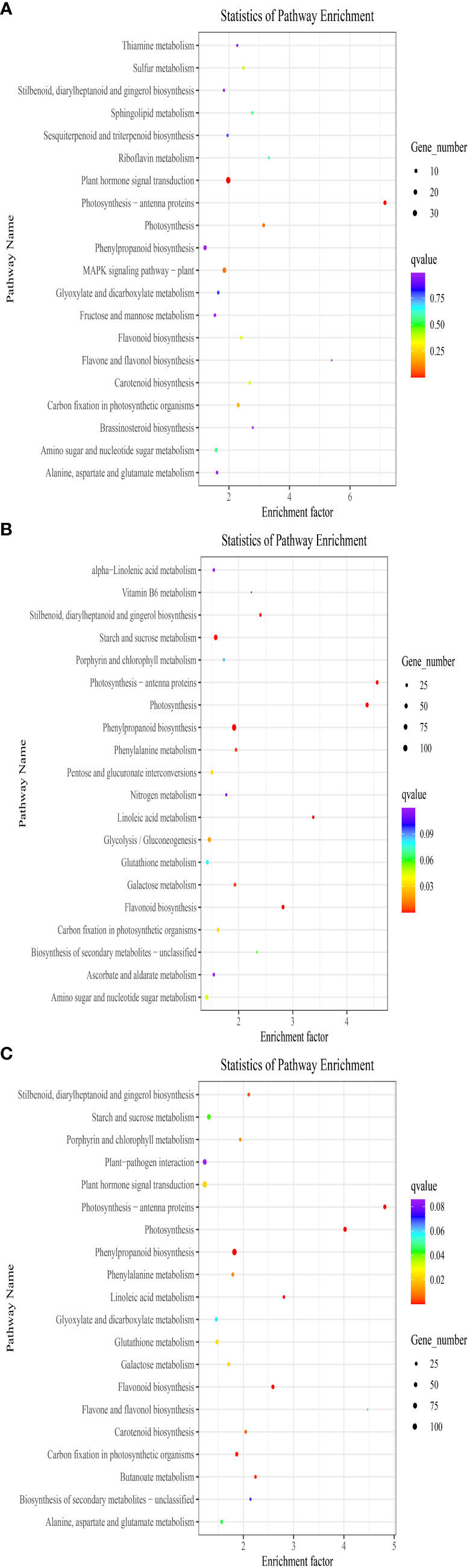
Scatter diagram of biological pathway enrichments of differently expressed genes between DP vs BP **(A)**, BP vs SP **(B)** and DP vs SP **(C)**.

The DEGs were analyzed using KEGG. As illustrated in **Figure 2A**, between DP and BP, the DEGs were primarily enriched in phenylpropanoid biosynthesis, mitogen activated protein kinase (MAPK) signaling pathway-plant, amino sugar and nucleotide sugar metabolism, plant hormone signal transduction, and alanine, aspartate, and glutamate metabolism. The DEGs between BP and SP were greatly enriched in phenylpropanoid biosynthesis, glycolysis/gluconeogenesis, amino sugar and nucleotide sugar metabolism, starch and sucrose metabolism, flavonoid biosynthesis, and other pathways (**Figure 2B**). Between DP and SP, the enriched DEGs were essentially found in carbon fixation in photosynthetic organisms, plant–pathogen interaction, starch and sucrose metabolism, flavonoid biosynthesis, phenylpropanoid biosynthesis, plant hormone signal transduction, etc. (**Figure 2C**).

### 3.3 Plant hormone signal transduction and transcription factor was activated

**Figure 3:**
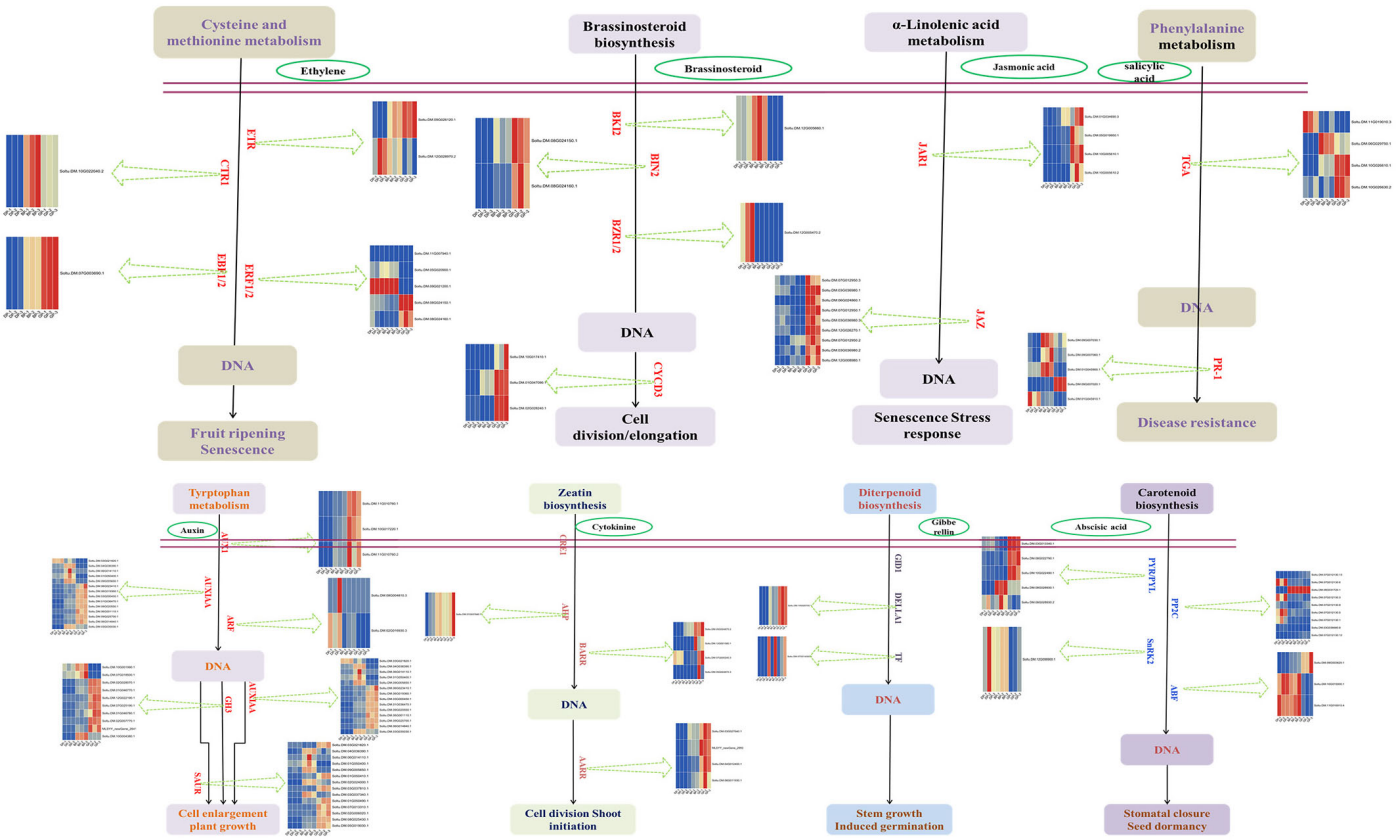
Heatmap of quantitative real-time reverse-transcription PCR (qRT-PCR) analysis in plant hormone signal transduction.

Transcriptome analysis showed that the plant hormone signal transduction pathway in the tuber was significantly activated during sprouting. From dormancy period to budding period, the expression of related genes in auxin and ethylene signaling pathways was up-regulated (**Figure S2A**). From budding period tonsprouting period, more signaling pathways were activated, mainly auxin, cytokinine, abscisic acid, brassinolide and jasmonic acid signaling pathways (**Figure S2B**). To verify the RNA-seq results, we used qRT-PCR (**Figure 3**). Interestingly, gene expression in ethylene signaling pathway is down regulated. In addition, many transcription factors also were activated, of which ethylene responsive factor (ERF), myeloblastosis-related (MYB), basic helix-loop-helix (bHLH), and WRKY families were the most in number (**Figure S3**).

### 3.4 Analysis of DAMs

**Figure 4:**
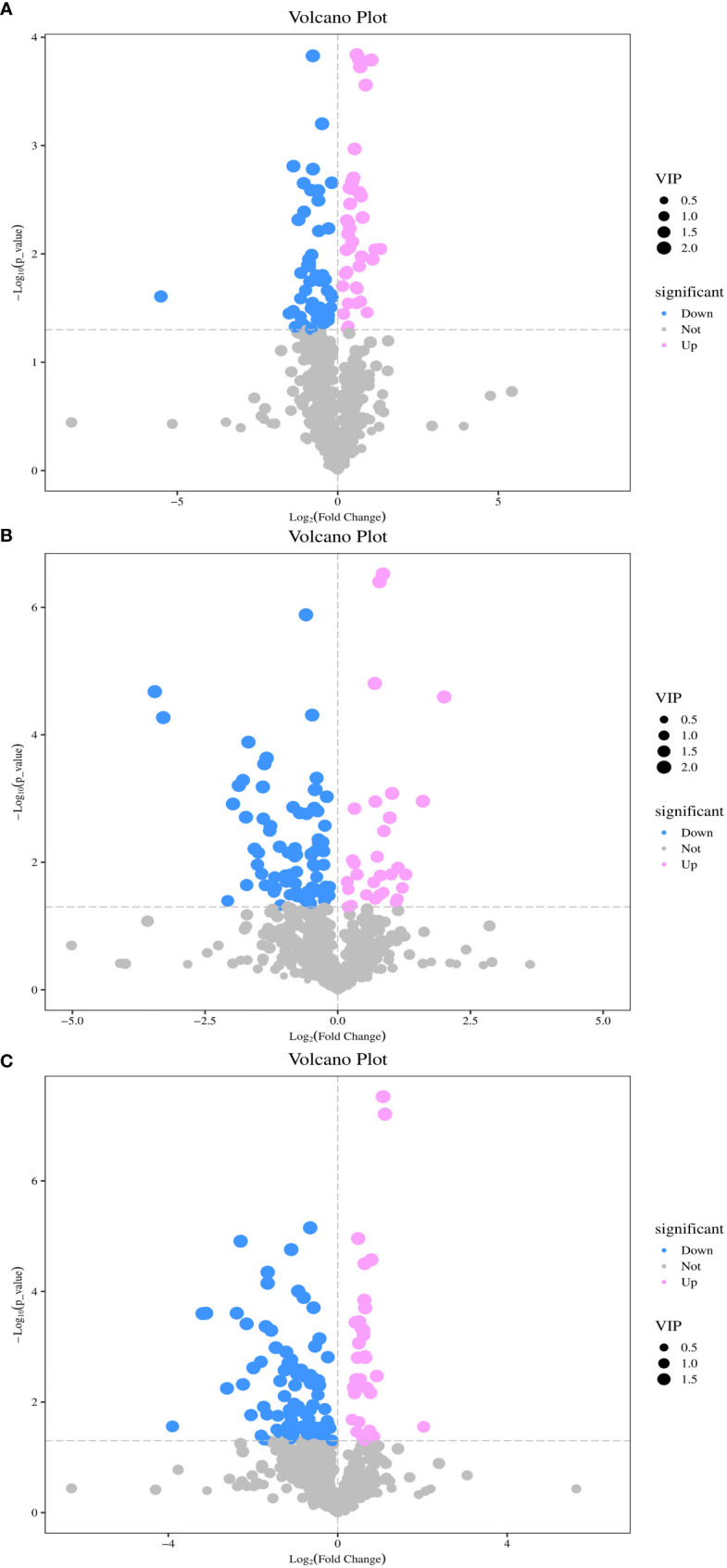
Volcano plot of differentially metabolites between DP vs BP **(A)**, BP vs SP **(B)** and DP vs SP **(C)**.

Univariate and multivariate statistical analyses were conducted for each comparison group to identify DAMs. As shown in **Figure 4A**, a total of 96 DAMs were detected between DP and BP, of which 39 were upregulated and 57 were downregulated. There were 117 DAMs between BP and SP, of which 85 were downregulated and 32 were upregulated (**Figure 4B**). When comparing DP and SP, 130 DAMs were detected, of which 36 were upregulated and 94 were downregulated (**Figure 4C**).

### 3.5 Statistical analysis of DAM analysis

**Figure 5:**
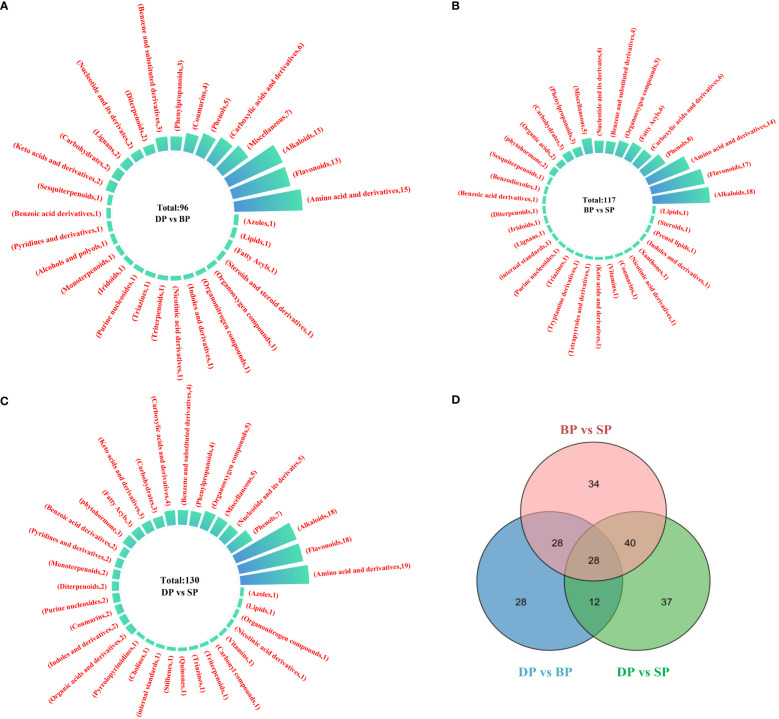
Statistics of differentially accumulated metabolites between DP vs BP **(A)**, BP vs SP **(B)** and DP vs SP **(C)**. Venn diagram between DP vs BP, BP vs SP and DP vs SP **(D)**.

During tuber sprouting, the main DAMs were amino acids and their derivatives, alkaloids, and flavonoids. Of the DAMs of DP and BP, the majority were amino acids and their derivatives, with 15 species, followed by flavonoids and alkaloids, with 13 species (**Figure 5A**). Of the DAMs of BP and SP, alkaloids were the highest, with 18 species, followed by flavonoids and alkaloids, with 17 and 14 species, respectively (**Figure 5B**). Of the DAMs of DP and SP, there were 19 alkaloids and 18 flavonoids and alkaloids (**Figure 5C**). A Venn diagram of the DAMs analysis indicated that 151 genes were shared across the three groups of paired comparisons. The results illustrated that 56 common DAMs appeared in the DP vs SP and DPvs BP. 68 DAMs were shared between DP vs SP and BP vs SP, and 40 DAMs were shared between DP vs BP and BP vs SP (**Figure 5D**).

### 3.6 KEGG enrichment analysis of DAMs

**Figure 6:**
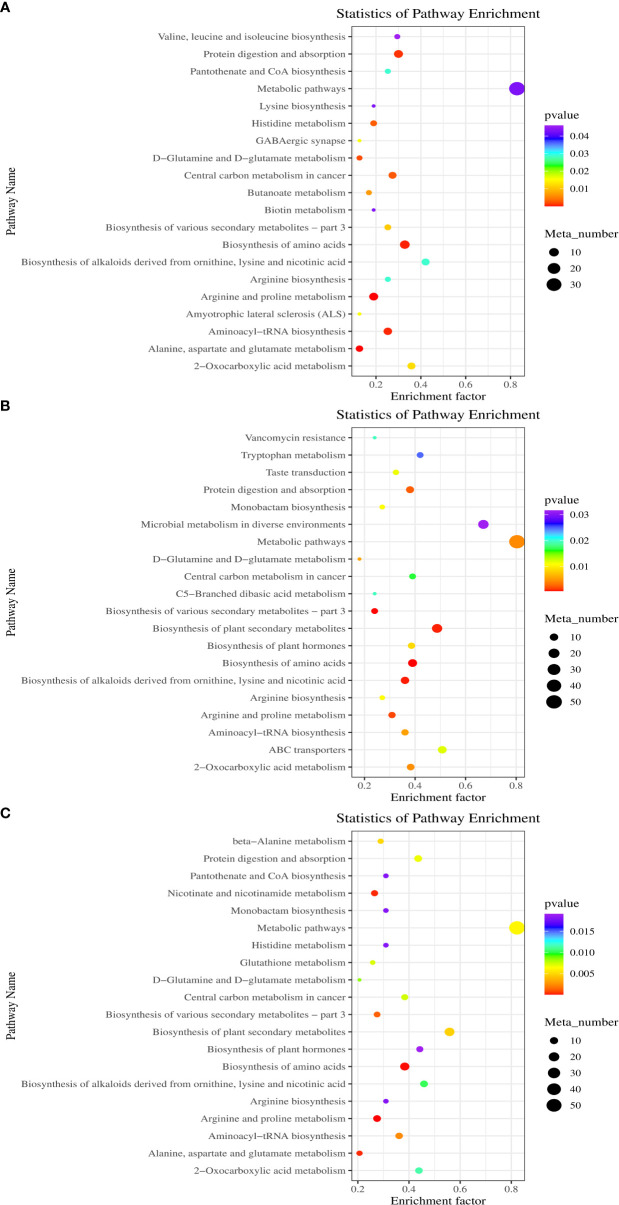
Scatter diagram of biological pathway enrichments of differently metabolites between DP vs BP **(A)**, BP vs SP **(B)** and DP vs SP **(C)**.

**Figure 6A** displays the KEGG enrichment analysis of all DAMs. The findings indicated that between DP and BP, the differential metabolites were largely enriched in arginine and proline metabolism, amino acid biosynthesis, aminoacyl-tRNA biosynthesis, alkaloids derived from ornithine biosynthesis, protein digestion and absorption, lysine and nicotinic acid, etc. The DAMs between BP and SP are described in **Figure 6B**. The DAMs were mainly concentrated in amino acid biosynthesis, alkaloids derived from ornithine biosynthesis, plant secondary metabolite biosynthesis, lysine and nicotinic acid, ABC transporters and protein digestion and absorption, and microbial metabolism in diverse environments. Between DP and SP, the DAMs were primarily related to amino acid biosynthesis, arginine and proline metabolism, alkaloids derived from ornithine biosynthesis, plant secondary metabolite biosynthesis, lysine and nicotinic acid and nicotinate and nicotinamide metabolism, etc. (**Figure 6C**).

### 3.7 Transcriptome and metabolome analysis

**Figure 7:**
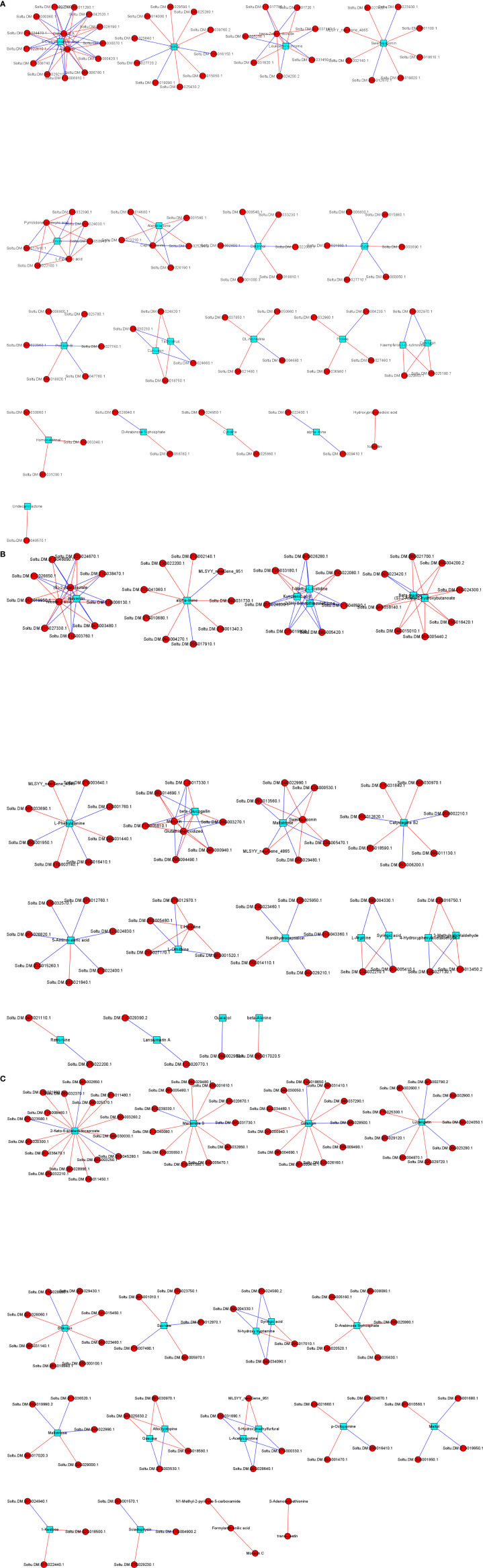
The regulation network of different genes and different metabolites between DP vs BP **(A)**, BP vs SP **(B)** and DP vs SP **(C)**.

Correlation analysis of the DAMs and DEGs in each group was performed to analyze the regulatory network. Several genes were found to play key roles in positively or negatively regulating multiple metabolites. For instance, 2-keto-6-acetamidocaproate was significantly correlated with 16 DEGs (13 positively correlated and 3 negatively correlated), macamide B and galangin were significantly correlated with 11 DEGs (9 positively correlated and 2 negatively correlated), and luvangetin was markedly correlated with 9 DEGs (5 positively correlated and 4 negatively correlated). More regulatory networks are shown in **Figures 7A–C**.

## 4 Discussion

Potato tubers are used as food or seed, and according to the purpose, sprouting needs to be either impeded/ceased (i.e., for industrial processing) or accelerated (i.e., for seed tubers) (Sonnewald, 2001). In-depth study of potato sprouting mechanisms can help achieve fine control of this biological process. In the present work, several DEGs were screened during tuber sprouting. These DEGs were accumulated in the MAPK signaling pathway and in hormone signal transduction. According to previous studies (Suttle et al., 2012; Sonnewald and Sonnewald, 2014), phytohormones serve as the most efficient and significant endogenous regulators in tuber dormancy and sprouting. Although the initiation and maintenance of tuber dormancy can be promoted by ethylene, its role is not completely understood. Studies have confirmed that ethylene is involved in the first phase of dormancy maintenance (Korableva and Platonova, 1995; Suttle, 2004). After tuber dormancy ends, gibberellins (GA) play a key role in stimulating the active growth of sprouts (Claassens and Vreugdenhil, 2000). However, sprout growth can be inhibited by ethylene and ABA (Sadawarti et al., 2016). High levels of synthetic exogenous auxins may prevent eyes from growing to some extent, whereas low levels of auxins may activate bud growth (Suttle, 2007). The function of jasmonic acid in tuber sprouting is also concentration-dependent (Platonova et al., 2010). Cytokinins (CK) can efficiently regulate dormancy and tuber sprouting. CK promotes the shift from tuber dormancy to its activation and bud growth (Suchova et al., 1993). Different phytohormones regulate tuber sprouting and dormancy (Aksenova et al., 2013). It has been reported that the interaction between gibberellins and cytokinins has an effect on controlling tuber sprouting (Alexopoulos et al., 2007). Interactions among various hormone groups can be promptly coordinated. However, the mechanisms of phytohormone coordination during tube sprouting need further exploration.

Phytohormones regulate sprouting by controlling various metabolic pathways in tubers (Boivin et al., 2020). During sprouting, potato tubers initially act as a source organ to support the growth of germinated sprouts (Sonnewald, 2001). In addition, the degradation of protein and starch is initiated, and amino acids and soluble sugars are produced (Hajirezaei and Sonnewald, 1999). In this study, metabonomic analysis demonstrated that several differential metabolites were enriched in the amino acid synthesis pathway. This result indicates that numerous amino acids are synthesized during tuber germination (Michaud et al., 2006). In addition, the results of metabonomics revealed that several alkaloids were accumulated during tuber sprouting. In solanaceous plants, such as eggplants and potatoes, alkaloids are present as secondary metabolites (Deng et al., 2020). The accumulation of alkaloids in tubers plays a dual role. On the one hand, GAs are natural toxicants, threatening food safety and human health (Yamashoji and Matsuda, 2013); on the other hand, GA production is a defense mechanism against organisms such as fungi, insects, and viruses (Sengul et al., 2004). During tuber sprouting, many flavonoids were detected. Flavonoids exhibit health-promoting activities, which are closely related to their antioxidant activity, via cell signaling, membrane characteristic mechanism, or pro-oxidative activity (Mein et al., 2008). In conclusion, metabonomics indicated that during tuber sprouting, amino acids, alkaloids, and flavonoids are accumulated in high amounts.

Metabolites are produced as part of the cellular biological regulation process. Their accumulation exerts a significant impact on regulating plant development and growth (Pichersky and Lewinsohn, 2011; De Luca et al., 2012). Various endogenous and exogenous factors influence the accumulation of metabolites. Metabolomics integrated with transcriptomics is a key technical approach to analyze functional genes and understand the corresponding metabolic pathways (Matus, 2016; Wang et al., 2017; Dong et al., 2019). According to collected data from metabolomic and transcriptomic analysis, several DAMs are modulated by a range of genes during tuber germination. While several DEGs are significantly increased in the MAPK signaling pathway and in hormone signal transmission, DAMs are mainly enriched in alkaloids derived from ornithine biosynthesis, amino acid biosynthesis, lysine, and nicotinic acid. Therefore, we believe that during stem germination, the initial phenomena are the production of plant hormones and the activation of MAPK signal transduction, which are followed by the activation of various metabolic pathways in the tuber, resulting in the production of amino acids, alkaloids, flavonoids, and other substances.

## 5 Conclusions

In the present study, a large number of DEGs were screened during sprouting of tubers. At different stages of potato sprouting, there are significant differences in the number of differentially expressed genes. But these DEGs were enriched in plant hormone signal transduction and MAPK signaling pathway-plant. In addition, transcription factors such as ERF, MYB, bHLH, and WRKY significantly upregulated during tuber sprouting, and they may directly interact with structural genes to exert their functions under hormone regulation. The results of metabonomic analysis demonstrated that several differential metabolites were enriched in the amino acid synthesis pathway, which shows that a large number of amino acids are synthesized during tuber germination. Metabonomics also indicated that several alkaloids were accumulated during tuber sprouting. The integrated analysis of metabolome and transcriptome suggested that this complex regulatory network comprises different genes and metabolites.

## Data availability statement

The data presented in the study are deposited in the the SRA database repository, accession number PRJNA1044237, https://www.ncbi.nlm.nih.gov/sra/PRJNA1044237.

## Author contributions

XZhe: Writing – original draft, Writing – review & editing. ML: Funding acquisition, Project administration, Writing – review & editing. XZha: Investigation, Methodology, Writing – original draft. JC: Project administration, Resources, Writing – review & editing. XG: Methodology, Software, Writing – review & editing. SL: Formal analysis, Writing – review & editing. JT: Data curation, Formal analysis, Writing – review & editing. ST: Resources, Supervision, Visualization, Writing – review & editing.
