# Supplementary material for: Unraveling the mechanism of potato (Solanum tuberosum L.) tuber sprouting using transcriptome and metabolome analyses
Source: Front Plant Sci. 2024 Jan 5;14:1300067. doi: 10.3389/fpls.2023.1300067 (PMC10796687; doi:10.3389/fpls.2023.1300067)
Supplement: Supplementary file 1 [file DataSheet_1.pdf]

Supplementary Materials

Supplementary Fig. 1 Changes of bud length during tuber sprouting.

Supplementary Fig. 2 Changes in plant hormones during tuber sprouting between DP vs BP (A) and BP vs SP (B).

Supplementary Fig. 3 Changes in the number of transcription factor families during tuber sprouting.

Supplementary Table.1 qRT-PCR primers.

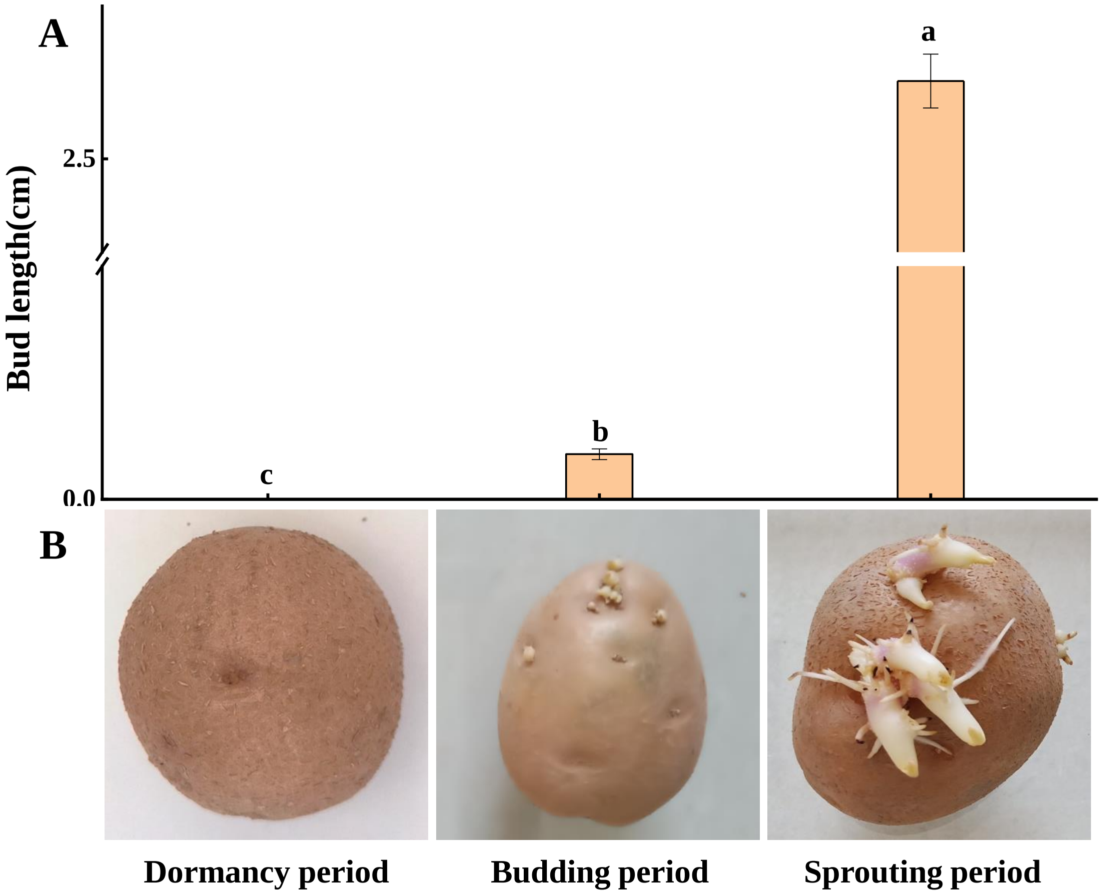

Supplementary Fig. 1

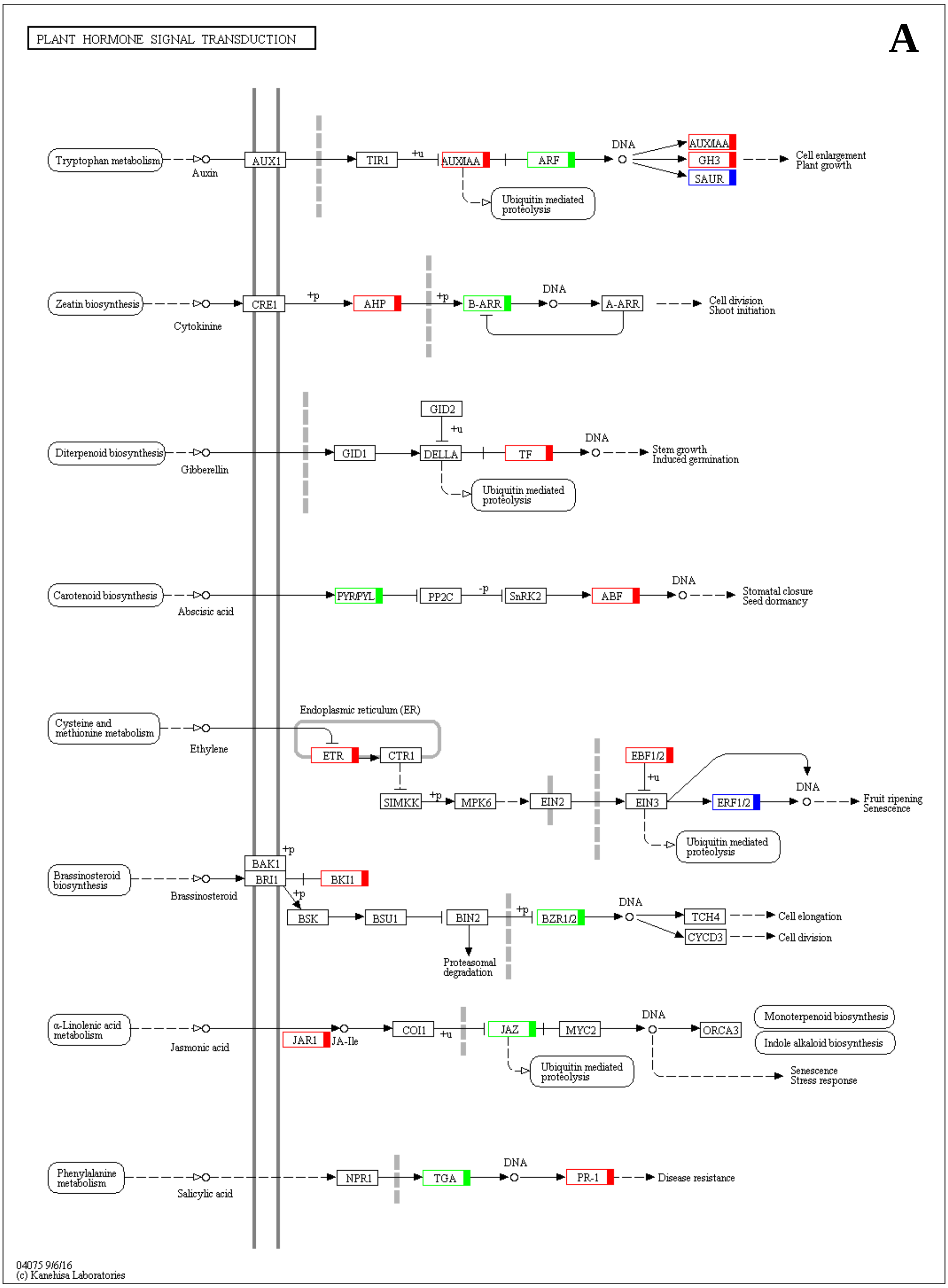
PLANT HORMONE SIGNAL TRANSDUCTION
A
Tryptophan metabolism
Auxin
AUX1
TIR1
+u
AUX/IAA
ARF
DNA
AUX/IAA
GH3
SAUR
Cell enlargement
Plant growth
Ubiquitin mediated proteolysis
Zeatin biosynthesis
Cytokinine
CRE1
+p
AHP
+p
B-ARR
DNA
A-ARR
Cell division
Shoot initiation
GID2
+u
Diterpenoid biosynthesis
Gibberellin
GID1
DELLA
TF
DNA
Stem growth
Induced germination
Ubiquitin mediated proteolysis
Carotenoid biosynthesis
Abscisic acid
PYR/PYL
PP2C
-p
SnRK2
ABF
DNA
Stomatal closure
Seed dormancy
Endoplasmic reticulum (ER)
Cysteine and methionine metabolism
Ethylene
ETR
CTR1
EBF1/2
+u
SIMKK
+p
MPK6
EIN2
EIN3
ERF1/2
DNA
Fruit ripening
Senescence
Ubiquitin mediated proteolysis
+p
BAK1
Brassinosteroid biosynthesis
Brassinosteroid
BRI1
BKI1
+p
BSK
BSU1
BIN2
+p
BZR1/2
DNA
TCH4
CYCD3
Cell elongation
Cell division
Proteasomal degradation
α-Linolenic acid metabolism
Jasmonic acid
JAR1
JA-Ile
COI1
+u
JAZ
MYC2
DNA
ORCA3
Monoterpenoid biosynthesis
Indole alkaloid biosynthesis
Ubiquitin mediated proteolysis
Senescence
Stress response
Phenylalanine metabolism
Salicylic acid
NPR1
TGA
DNA
PR-1
Disease resistance
04075 9/6/16
(c) Kanehisa Laboratories

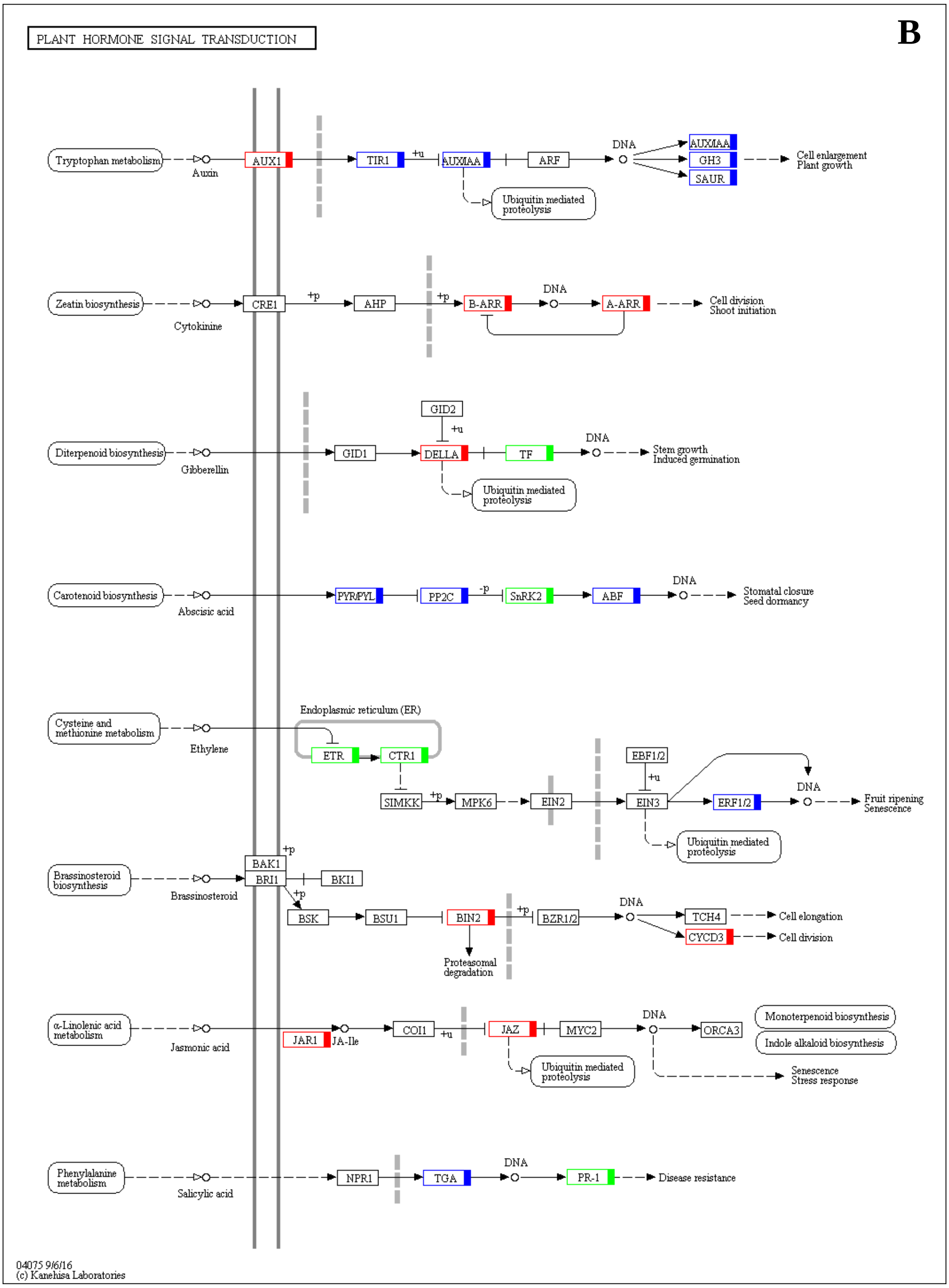

Supplementary Fig. 2

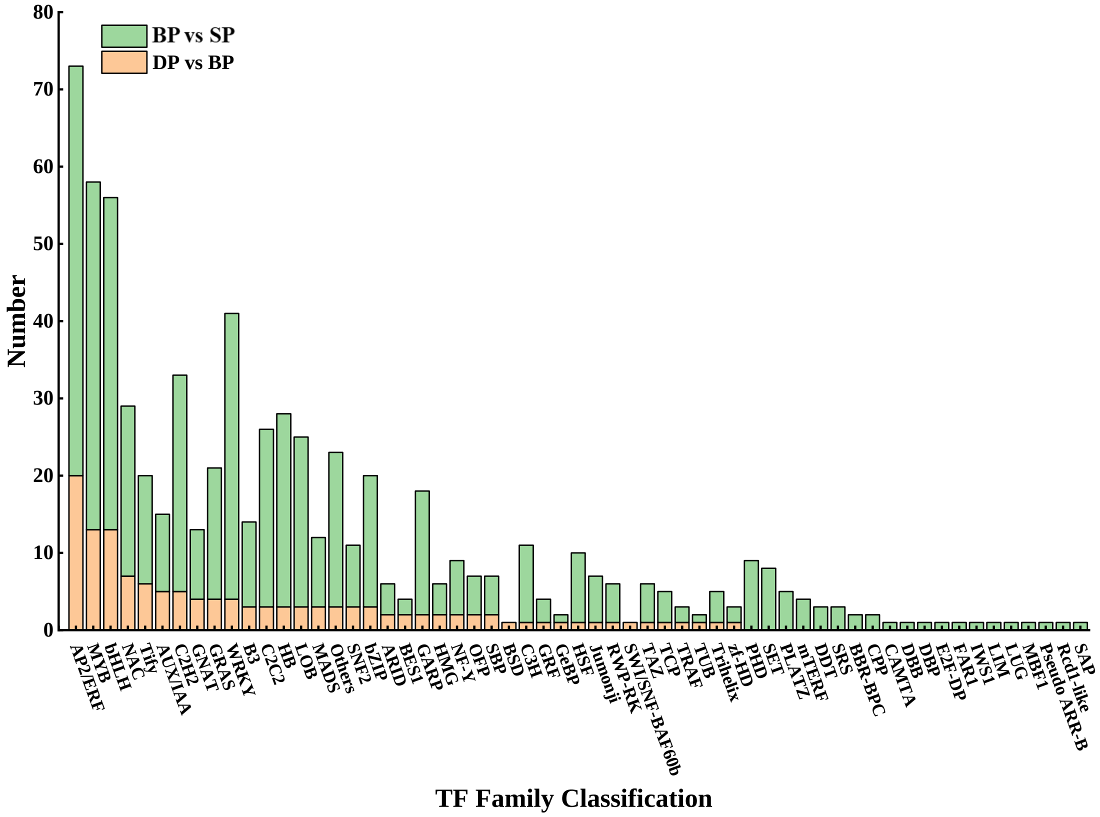

Supplementary Fig. 3

Gene ID	Primer-F (5'-3')	Primer-R (5'-3')	Product size
Soltu.DM.11G010760.1	TTCCTGCCTTAGCACACA	TACAACCCAACCCACCTT	100
Soltu.DM.10G017220.1	TTATGTAGAAATGGAGA	ATTGAGAAAATGAGTATG	158
Soltu.DM.11G010760.2	GCACTCAGGACCAACAAC	TCCAAAAGCCCAATACAC	202
Soltu.DM.02G018730.1	GGAAGTATGAATCAATGC	AGTTACAACAAAAGGTGG	200
Soltu.DM.02G018730.2	TAGTTGGCTAAGTTGTTT	GTGAGTTGATCTGGAAGA	234
Soltu.DM.03G021820.1	GGATCGGACACAAACACC	CCCTGAAGCACCACAAGT	336
Soltu.DM.04G036390.1	GAAGCAAATACTAAAAAG	CCAACGTAAACTACAAAA	114
Soltu.DM.06G014110.1	ATCTACCTCACATCCATT	TCTTCTTCATTACCTTTC	196
Soltu.DM.01G050400.1	CTTCTTCATCTAAACGCA	GAATAAAAACTCATCGCA	200
Soltu.DM.09G005650.1	GCCAAAATAAATGGGAAA	GCCTTGAAGAGTGGATGC	202
Soltu.DM.06G023410.1	TATCGGAGAAAGAACAAT	TCACTAAACATCAGCCAG	254
Soltu.DM.06G019360.1	AATGGAGTAGTGGGGTGG	TTTGGAGCAAGGTGTTAG	232
Soltu.DM.03G000450.1	TGGAGCCCCTTTTCTTAG	AAATCGCCTTCTTTATCT	168
Soltu.DM.01G036470.1	TGAAGGAGAAGAAGAATA	TGTAAAAGAGCTGAACAT	290
Soltu.DM.09G020550.1	ATTAGGTTTGCCTGGGAT	TTGGTGGAGGAGGTGAGT	122
Soltu.DM.06G001110.1	CTAGGGTTGCCTGGGGAA	GCTGGGGCTTTGAGTGTT	146
Soltu.DM.09G025700.1	AATGATGATATTTCTTGT	TTCTTGGTAACTTTTGTA	276
Soltu.DM.06G014840.1	GTGAGAAGGGAGAAAAAA	TGGTCAAAGAATCAGAGA	120
Soltu.DM.03G035030.1	TCTCAAAGTGAAAGCAAG	TGGAACATCTCCAACAAG	102
Soltu.DM.08G004810.3	TCAGTCGCTACTCCTCTC	CCTTCTGTCTCATCCAAA	290
Soltu.DM.02G016930.3	GCTCGTTATAGCAAACCG	ATCTGACCAAAAGGCACC	298
Soltu.DM.10G001990.1	TTACACCTTTATTCCCAC	TCAAGATTACATTTTCCC	278
Soltu.DM.07G018500.1	GCTTATACTCTCATACCT	ACATTCTTTCTACACACA	254
Soltu.DM.02G028070.1	CTAAGGTCCCCGTTGTCA	AGGCGTCTTGGTTTCTGC	284
Soltu.DM.01G046770.1	CCTCACCTACCAATCTCG	GCATTCTCTACGGCTCTC	232
Soltu.DM.12G022190.1	CGTTGGTGGATTTAGTTG	GGCGATAGACGCTGTTTA	408
Soltu.DM.07G025190.1	ATTCTTGCCTGTTCATCG	TAGCACCACATTCTTCCT	244
Soltu.DM.01G046780.1	TACTTTCATCTCCTTTGG	ACTTTCTCCTGTAATCCC	300
Soltu.DM.02G007770.1	CATCTCATCCCATCTCTG	TCTTGGTTTCTGCTTTTA	198
MLSYY_newGene_2641	AATACTCAAACCAAATCA	CATACCTGTCACAATCAC	128
Soltu.DM.10G004380.1	TACTATGGTTCCTCTTGC	TTGTTTTGTATTGTTTGC	284
Soltu.DM.03G021820.1	CGGACACAAACACCATCC	TCCTCACCTTGACCCACC	138
Soltu.DM.04G036390.1	GTTCGAGTATAGGAAAGA	TCACATGGAATTGTAAGT	220
Soltu.DM.06G014110.1	ATCTACCTCACATCCATT	TCTTCTTCATTACCTTTC	196
Soltu.DM.01G050400.1	CTTCTTCATCTAAACGCA	GAATAAAAACTCATCGCA	200
Soltu.DM.09G005650.1	GCCAAAATAAATGGGAAA	GCCTTGAAGAGTGGATGC	202
Soltu.DM.01G050410.1	ATTAGAAAATCAAACAAG	GGATGAGTCAAAAAAGAA	194
Soltu.DM.02G024000.1	TGTTTATGTTGGAGAAAA	ACAAGGAATAGTAATGCC	136
Soltu.DM.03G037810.1	TAGTAGTGGTAATGGCGG	CTTGAAATGATTGGTGGG	182
Soltu.DM.03G037340.1	GCCGTTATGACACCAAGA	CTCCCACCAGCACAAGTA	230
Soltu.DM.01G050490.1	ACCAGAGATATTCCCAAG	ATAGTCACACCACCCATT	164
Soltu.DM.07G013310.1	ATTAATGTGTGTTGTGAT	CTAAGGTAAGTTGTAGGA	134

Soltu.DM.02G006020.1	AAGAAGGGGAGAGGAGAT	CTTGGAAAAAGCAATAAA	226
Soltu.DM.08G025430.1	AAGAACCGATTGCTCCAA	TCCACCTCAGCTCTCCAC	276
Soltu.DM.05G019030.1	CAACTCTCTTCGTCCTCG	CATCCTCATCAACCCCTA	134
Soltu.DM.01G037840.1	GGAACAAATCCAAAGAAA	AAGAAAAGGGACACAACT	108
Soltu.DM.05G024870.2	CAAAGTCTTGCTACCCTC	TTCCCTTGCATATTCATA	260
Soltu.DM.12G001580.1	GTTAGCAGACCAGAGAGA	CATTGTGAACGAGACCAT	248
Soltu.DM.07G000240.3	ATGAAAATGGAAATGACG	TGTTGGGTTTGAACTGAA	238
Soltu.DM.05G024870.3	GGTTTGGTCGGTGGAGCT	GCAAGACTTTGTGCGGGT	232
Soltu.DM.03G027640.1	TACCCGTTGTTATTATGT	CTCTGTTTCTTTGTTGTC	164
MLSYY_newGene_2993	TGATTTCCGTCTCTTTTG	CCCCCTCACCTCTTAGTA	248
Soltu.DM.04G012400.1	AATCCCCCTTGTGTTTCT	ATCTTCTGCCCCTTCTTC	216
Soltu.DM.06G011930.1	CCTCATGTCTTAGCTGTT	ACGATTCCTTTATTTTCT	244
Soltu.DM.10G020700.1	GGCTTGCGAAGGAACTAA	GTGAAGAGCGGGCGACTA	216
Soltu.DM.07G014300.8	CCCTATCTATGATTCGTT	CCTTATTATTGTCCTCTG	122
Soltu.DM.03G013340.1	TTACATTACCACACCCAC	TTACCTGAACCTCCCTTA	196
Soltu.DM.08G022790.1	TATCATTGGCGGAGAACA	TATCAGCGAAAAGGCGTG	158
Soltu.DM.10G022490.1	TACCCGATTCTGTTTCCC	AATGCTTATACGCCTGCG	144
Soltu.DM.08G028930.1	TTGTTACTTTGTTGAGGC	ACATTCTGTCGATAGGCT	104
Soltu.DM.08G028930.2	ACCACAAGCAAGGAGAGG	TTTGAGGTTACACCGAAT	240
Soltu.DM.07G012130.13	CGGAATGAAGACAAGAAG	GAATCAAAACAAAAGGCA	218
Soltu.DM.07G012130.6	CGGAATGAAGACAAGAAG	CAGAATCAAAACAAAAGG	220
Soltu.DM.06G031720.1	GATAACGCTGTGGAAAGT	GCAAAATGAAGGATGAAT	253
Soltu.DM.07G012130.3	CGTTTCTGGTGTTCTTGC	TCGTCTTCTTTTGCTCGG	108
Soltu.DM.07G012130.8	AGGTTAAAGTTGGGAGAA	CAGAATCAAAACAAAAGG	246
Soltu.DM.07G012130.5	CGGAATGAAGACAAGAAG	GAATCAAAACAAAAGGCA	218
Soltu.DM.07G012130.1	CGGAATGAAGACAAGAAG	CAGAATCAAAACAAAAGG	220
Soltu.DM.03G036680.8	AACTGTTGGGTCCACTGC	CATAACTTCTGGTTCGGG	280
Soltu.DM.07G012130.12	CGTTTCTGGTGTTCTTGC	ACAGTCGTCTTCTTTTGC	112
Soltu.DM.12G006900.1	TAGTAATGGAGTATGCGG	ACAGTGGACTTTGGTTGA	256
Soltu.DM.09G003620.1	GGTTATCCGCCACTTCCA	TTCGCCCACCTCTCATTC	268
Soltu.DM.10G015000.1	ACACTGCCACAGACACTT	ACTCCTGCTCTTACCAAA	146
Soltu.DM.11G016910.4	ATGTAAGTGGGACTGGAG	CTTGTTGAAATGCTATGG	192
Soltu.DM.09G026120.1	TCTTTTTCCCAAACAAAC	TATCATCCTCTTACGCCT	274
Soltu.DM.12G028970.2	GTTCTGATGCTTCCTTCG	TTCTGCTTCTCTTCTCGC	186
Soltu.DM.10G022040.2	AATGGCTCTGATGTTGCT	TGGTTTGCGTTTGTGAAG	300
Soltu.DM.07G003690.1	GGGCTTCTCCCACTTTTG	ACCCTCCTGCATCCATCA	152
Soltu.DM.11G007940.1	ATAGTGTTAGGGTTTGGT	TTTTCTTCCTTCTTTTTA	250
Soltu.DM.05G020900.1	CAACAACATCAAAAGCAA	GAGAAGAGACCAAGGACC	260
Soltu.DM.09G021200.1	CTATACTTAACTTTCCTG	CTACTCACACTCTTTCTC	130
Soltu.DM.08G024150.1	AAATGGGCAAAGAGTGTG	TATCCGATGACGACGAAG	206
Soltu.DM.08G024160.1	GCGATGATTTCTACAACG	AACCGACTCTAACAGGCT	256
Soltu.DM.12G005660.1	GGCACTTACTTCCTCTTC	TTCCCTTAGCATCACAAC	162
Soltu.DM.08G024150.1	AAATGGGCAAAGAGTGTG	TATCCGATGACGACGAAG	206
Soltu.DM.08G024160.1	GCGATGATTTCTACAACG	AACCGACTCTAACAGGCT	256

Soltu.DM.12G005470.2	CCGATGGAGATTGGAGGC	TCAGCACGGGATGGACTG	146
Soltu.DM.10G017410.1	GTTGATTGCCTTTACTGT	TTTCTAGACCCATCTGAG	218
Soltu.DM.01G047090.1	CTGATTGTAGATTCGTAC	TTATTCACATTCTCCTTG	148
Soltu.DM.02G028240.1	TGGATTGGATGTTGAGGG	ACAAAAGGGGAACTTGGG	192
Soltu.DM.01G034690.3	TCAAGACAAAGGGAGGTT	ACACAAGAGATGGCAGTA	164
Soltu.DM.05G019950.1	ATTATTCCCAAACACCAG	GCCATCTACATTTTCCAC	238
Soltu.DM.10G005610.1	TACTTATGCAGTGTTACC	CTTTCTCAGTGTTCTTGT	278
Soltu.DM.10G005610.2	TACTTATGCAGTGTTACC	AAATCTTTCTCAGTGTTC	282
Soltu.DM.07G012950.3	GATTCAACTGGTTCTACT	TTGTTTTCTTCCTTCTTA	266
Soltu.DM.03G036980.1	CTTGTTGAGTCAGTTTAT	TGTTCTTCTTTAGTTAGA	156
Soltu.DM.06G024860.1	TGGTTCTGTCTGTGTTTA	TGTTGTTTCATTCGTATT	142
Soltu.DM.07G012950.1	AAGGCACAGATGACGATA	AAACTGGGAACAACCAAA	158
Soltu.DM.03G036980.3	CACCACAGCAACAACACC	GCCAAATACTGCCATATA	254
Soltu.DM.12G026270.1	CAATGCCACAGCCTTCTG	CAATGCCACAGCCTTCTG	152
Soltu.DM.07G012950.2	CAAGGGAAACAACACACA	AGACACTCAAAAGGGGAA	140
Soltu.DM.03G036980.2	CACCACAGCAACAACACC	GCCAAATACTGCCATATA	254
Soltu.DM.12G008980.1	AACAGAGCAAGACCAGAA	CACTTTACCCCCATAGAA	130
Soltu.DM.11G019010.3	CTTGTGGCTTGGTGGATT	GCCTGTTGGGATGACTGT	120
Soltu.DM.06G029750.1	TATGCTGTTTTTAGGGGG	TCTTGGGGTCTGTTGGTA	246
Soltu.DM.10G026610.1	AACACACCAAACACATCA	CACCAATCCACATAAAAC	204
Soltu.DM.10G026630.2	CTATGGGGAAATTAGGGA	TCAAACAACTGAACGAGC	130
Soltu.DM.09G007030.1	TATTTCACTCTTGTGATG	TAGTTGTAGTTTGGCTTC	258
Soltu.DM.09G007060.1	GGCAACCCGAGCACAAAA	CGAGCCCGACCACAACCT	246
Soltu.DM.01G045900.1	GTGTTCTTGGTTTTAGGC	ATCGTTCCATTTTAGGGG	126
Soltu.DM.09G007020.1	TATTTCACTCTTGTGATG	AGTTGTAGTTTGGCTTCT	258
Soltu.DM.01G045910.1	TCTTAACGCTCACAATGC	AATCACGCCAGACTACCT	290
ef1AB061263	ATTGGAAACGGATATGCTCCA	TCCTTACCTGAACGCCTGTCA	101

Supplementary Table. 1
